# Analysis of Chemical Composition of Different Irreversible Hydrocolloids

**DOI:** 10.3390/dj6030037

**Published:** 2018-08-02

**Authors:** Antonio Ricardo Borges de Olival, Nilton Luiz da Penha Junior, João Victor Frazão Câmara, Ana Clara Corrêa Duarte Simões, José Rodolfo Estruc Verbicário dos Santos, Sonia Groisman

**Affiliations:** 1 Faculty of Dentistry, Federal Fluminense University, Alameda Barros TerraStreet—Centro, Niterói-RJ 24020-150, Brazil; dentista.ricardo@gmail.com (A.R.B.d.O.); nlpj@hotmail.com (N.L.d.P.J.); 2 Faculty of Dentistry, Federal University of Rio de Janeiro, 255th Professor Rodolpho Paulo Rocco Street—Ilha do Fundão, Rio de Janeiro-RJ21941-913, Brazil; jvfrazao92@hotmail.com (J.V.F.C.); anaclara13simoes@gmail.com (A.C.C.D.S.); 3 Faculty of Dentistry, Educational Foundation Serra dos Órgãos, 111th Alberto Tôrres Av., Alto, Teresópolis-RJ 25964-004, Brazil; jvfrazao92@gmail.com

**Keywords:** irreversible hydrocolloids, Cadmium, toxic, contamination

## Abstract

Irreversible hydrocolloids (IR) is a dental impression material commonly used in Brazilian and European dental practice because it is inexpensive, easy to handle, has good reproductive detail and is comfortable for the patient. This research aimed to analyze the chemical composition of eight different IRs for dental use. A sample of 0.2 g was weighed and transferred to a Teflon beaker moistened with drops of distilled or deionized water; 5 mL of nitric acid was added until total solubility of the sample; the solution was transferred to a 100 mL volumetric flask, the volume was filled with distilled or deionized water and homogenized. Thirty-five chemical elements were found: Lithium, Beryllium, Boron, Sodium, Magnesium, Aluminum, Silicon, Phosphorus, Potassium, Titanium, Manganese, Cobalt, Nickel, Vanadium, Zinc, Rubidium, Arsenic, Iron, Copper, Strontium, Yttrium, Zirconium, Niobium, Molybdenum, Ruthenium, Cadmium, Tin, Antimony, Barium, Lanthanum, Cerium, Mercury, Lead, Thorium and Uranium. Only one of the samples contained no Nickel, Antimony and Lead; and Arsenic and Uranium were found in 2 samples. This study provided evidence of high toxicity of the IR brands, pointing out the need for better quality control of this product, in order to prevent health damage in dentists, prosthesis technicians and patients.

## 1. Introduction

Irreversible hydrocolloid (IR) is a dental impression material that is easy to handle, allowing good reproducibility detail, as well as being inexpensive and comfortable for the patient [1].

The biocompatibility tests, carcinogenic and mutagenic effects of dental materials are extremely important, since in dentistry different types of materials used for many surgical procedures may come into contact with cells of the oral mucosa, marginal gingival and/or dentin-pulp complex. Several studies have drawn attention to the relevance of checking the degree of cytotoxicity of dental materials, considering it an important step before its clinical use in the oral cavity [2,3]. Many substances such as Zinc, Cadmium, lead silicate and fluorides are added into some irreversible hydrocolloids brands, with the aim of improving their physical, chemical and mechanical properties, however the toxicity of these materials has become a concern [3].

Irreversible hydrocolloid intoxication can occur through the inhalation of dust by the patient and dental professional, and through accidental ingestion by the patient and absorption in the oral mucosa when repeated impressions are taken [4,5].

During an impression, the irreversible hydrocolloid comes into intimate contact, for about 2 min, with the oral mucosa, which is highly vascularized and has great absorption potential. Thus, the repetition of consecutive impressions could cause a certain degree of toxicity to the patient, depending on the composition of the material [6,7].

The most described products, in terms of toxicity, include: Cadmium, which is a pollutant of global concern due to its high toxicity even at very low concentrations, as well as its long half-life in humans, in a variation of 10 to 30 years it is still associated with the arising of neoplasms in the lung, prostate and testis and cardiovascular lesions such as atherosclerosis and hypertension [8]. 

Freitas, in 1980, reported that zinc concentrations in irreversible hydrocolloids were high, with values up to 6.05% by mass for the Jeltrate trademark. Zinc oxide acts as a bulking agent and influences its physical properties and gel setting time. Chronic oral exposure to zinc may result in microcytic or sideroblastic anemia, hypocupremia, neutropenia or pancreatic lesions; in addition, it causes teratogenic effects in humans [3].

Therefore, the purpose of the present study is to analyze the chemical composition of eight different irreversible hydrocolloids for dental use.

## 2. Material and Methods

Eight irreversible hydrocolloids brands were chosen and delivered to CETEM (Mineral Technology Center) at the Federal University of Rio de Janeiro, where the samples were analyzed using a technique for the extraction of metals. A sample of 0.2 g of each irreversible hydrocolloid brand was collected, to be assessed using the ICP-MS-model-7700, Agilent Brand, on 15 June 2015. After this process that lasted about 6 h, the sample was transferred to a 100 mL flask for digestion with nitric acid.

The acid digestion was performed using technology adapted by Arnaldo Alcover Neto, CETEM. All material was decontaminated with 10% HNO_3_ (the same acid used in the preparation of standards) followed by distilled or deionized water.

1. The Teflon beaker was moistened with drops of distilled or deionized water and was filled with 0.2 g of the sample;
2. 5 mL of nitric acid was added until total solubility of the sample;
3. The solution was transferred to a 100 mL volumetric flask, the volume was filled with distilled or deionized water and homogenized;
4. The elements were determined with the ICP-MS-model-7700—Agilent Brand;
5. A blank test was run in parallel using all reagents in the same amounts.

## 3. Results

In all the analyzed samples, 35 chemical elements were found: Lithium, Beryllium, Boron, Sodium, Magnesium, Aluminum, Silicon, Phosphorus, Potassium, Titanium, Manganese, Cobalt, Nickel, Vanadium, Zinc, Rubidium, Arsenic, Iron, Copper, Strontium, Yttrium, Zirconium, Niobium, Molybdenum, Ruthenium, Cadmium, Tin, Antimony, Barium, Lanthanum, Cerium, Mercury, Lead, Thorium and Uranium (Table 1, Table 2, Table 3 and Table 4). 

Only one of the samples had no Nickel, Antimony and Lead; Arsenic and Uranium were found in 2 samples. Fifty percent of the samples were contaminated with Mercury and all samples contained Beryllium, Cadmium and Copper.

Table 1 shows that the chemical elements with the lowest concentrations are Lithium (Li) and Beryllium (Be) whose values range from <0.104 ppb to 0.155 ppb and<0.125 ppb, respectively, in all the irreversible hydrocolloids evaluated. Sodium (Na) presented a concentration of 79,393 ppb in the irreversible hydrocolloid 5, followed by Magnesium (Mg) with 408,852 ppb in the irreversible hydrocolloid 2. In Table 2, the lowest reported concentration was 0.015 ppb related to Vanadium (V) in irreversible hydrocolloid 8, followed by Cobalt (Co) with 1 ppb in all irreversible hydrocolloids, with the exception of number 8 in which it was not identified. A high concentration of Zinc (Zn) was observed in irreversible hydrocolloid 5, corresponding to 575,658 ppb, followed by Iron (Fe) with 12,295 ppb in the same irreversible hydrocolloid.

Table 3 shows that the chemical elements with the lowest concentration are Cadmium (Cd), which ranges from <0.009 ppb to 1 ppb and Ruthenium (Ru) with <0.004 ppb in all irreversible hydrocolloids. At a high concentration, there is Niobium (Nb) with 420 ppb in irreversible hydrocolloid 5. In Table 4, Thorium (Th) and Uranium (U) are identified only in irreversible hydrocolloids 2 and 3 with a concentration of 1 ppb. There is a high concentration of Lead (Pb) of 354 ppb in irreversible hydrocolloid 5.

Therefore, based on the data analysis, irreversible hydrocolloid 5 presented significant concentrations of Zn, Fe, Nb and Pb.

## 4. Discussion

Irreversible hydrocolloid is one of the most accepted impression materials used in dentistry. The manufacturers produce irreversible hydrocolloid powder containing a lot of components for different purposes. However, these components can affect the ability of cells to reproduce. They may not be toxic enough to kill the cells but are toxic enough to inhibit cell growth or affect normal cell function less severely. It is important to highlight that while a single contact may not cause clinical symptoms, repeated contact with the material, which alters or affects the viability of cells, may result in a late or allergic toxic reaction [6]. 

Cadmium is a chemical element that has a long half-life (around 10 to 30 years) and it can be stored in the heart, spleen, testicles and pancreas, which characterizes it as highly toxic. As it is a polluting agent that affects society, Cadmium is the target of several studies [9].

In 2001, Braga et al. carried out a study aiming to histologically investigate the effects of cadmium ion on the submandibular gland of rats. Rats that were exposed to Cadmium for 6 months presented acini in the minor glands with a distorted shape and alteration in the shape of the nuclei. In addition, it was found that the striated, intercalated and interlobular ducts were disordered. When comparing the rats that were submitted to detoxification for six months with the control group their submandibular glands were equivalent histologically [10].

Pithon (2009) evaluated the cytotoxicity of four brands of irreversible hydrocolloid used in Dentistry: Jeltrate, Tropicalgin, Cavex Color change and Qualitygel. The material was handled following the manufacturer’s guidelines and added to silicon rings. Fibroblast cells L929 were used and the viable cell count was performed through a spectrophotometer at a wavelength of 492 nm. The results showed that all the irreversible hydrocolloids analyzed presented toxicity [11].

In 2010, Pithon conducted a new study evaluating the cytotoxicity of other irreversible hydrocolloid brands including Ava Gel, New Print, Kromopan and Hydrogum. The methodology was the same as that used in the previous study, so the supernatants were collected after 24, 48, 72 and 168 h. It was concluded that all irreversible hydrocolloids showed toxic potential to cells. The cell control group demonstrated greater viability followed by the negative control group, Hydrogum, New Print, Kromopan and Ava Gel [12].

In order for commercial products to reach the market at a more affordable cost, it is possible that steps in the process of removing impurities and decantation are skipped. However, this way of eliminating costs and/or maximizing profits can cause irreversible damage to the health of consumers and users. It is important to note that such chemical elements may have some quantitative increase when water is added. 

Dentists and dental auxiliaries may be exposed to high dust. It is recommended by the manufacturer that the material is agitated in the container before use. However, the powder spreads into the environment when the package is opened [11]. There is a scale which allows the classification of chemical elements according to their toxicity: 1. practically non-toxic; 2. slightly toxic; 3. moderately toxic; 4. very toxic; 5. extremely toxic and 6. super toxic. When this tool is used Barium sulfate (insoluble) is classified at level 1, lead salts and zinc oxides and salts are between levels 3–4, the other insoluble barium salts are between 3 and 5, soluble cadmium salts and fluorides are at level 5 and cadmium salts suspended in the air (inhalable) at level 6 [7].

Researchers in the dental area have been concerned with studying the physical and mechanical properties of dental materials, especially on the local and systemic biocompatibility of such materials [3]. Face masks reduce the aspiration of irreversible hydrocolloid powder, however, particles with diameters smaller than five micrometers cab cross it and can lead to damage of the oral tissues of dentists or dental technicians [5,7].

The deposition of inhaled particles of more than 10 micrometers in the respiratory tract occurs mainly in the upper respiratory part (above the larynx) and particles of 5 to 10 micrometers in aerodynamic diameter are deposited in the lower respiratory tract. Particles between two and a half and five micrometers are deposited in the conducting zone and bronchioles during normal nasal breathing [13,14].

Even if the face mask does not provide full protection to the professional, its use is recommended when handling irreversible hydrocolloid, as well as keeping the working environment clean and with adequate ventilation. Currently some commercial brands of irreversible hydrocolloid have printed on the package information about dust free material [13].

Despite the technological advances of the irreversible hydrocolloid companies, it is not possible to say that the use of irreversible hydrocolloid is totally safe for the professionals who manipulate it due to the shortage of studies on this subject. According to Buchan and Peggie significant care must be taken to ensure that the soluble lead salt, present in some irreversible hydrocolloid powders, is kept at a low and safe level, since it is up to the manufacturer to determine the amount required for its formulation. Besides, accidental intake of the material by the patient may also occur [9].

Lead is another element present in irreversible hydrocolloids. It is also absorbed by the body and then distributed to the blood, soft tissues and bones. The half life lasts between 25 and 28 days in the blood and 20 years in bone tissue. The literature describes some non-specific signs and symptoms of lead poisoning such as metallic taste, constipation, insomnia, irritability, muscle and joint pain, tremors, colic and gums with a bluish purple coloration. Nervous, hematological, cardiovascular and reproductive organs and systems, kidneys and fetuses are affected by specific symptoms [15].

Children were examined by Desres et al. (2005) and a neuromotor effect due to blood lead levels below 10 g/dL was reported. However, there are still no evident signs of lead poisoning in the initial phase, being asymptomatic until it evolves into a seizure or encephalopathy [16].

For Wani et al., inhaled lead intoxication occurs 1 to 100 times more than ingested intoxication, although no carcinogenic or fibrinogenic activity in dentists caused by the inhalation of irreversible hydrocolloid powder has been proven. The size and shape of the particles, as well as the long period for which they lie latent before the arising of signs and symptoms that suggest fibrotic disease, irreversible hydrocolloid powder aerosols should be considered as a potential risk and thus should be treated as such until proven otherwise [17]. 

## 5. Conclusions

All the samples had Beryllium, Cadmium and Copper above the normality patterns presenting a toxic potential to the cells.

It was highlighted that taking care to handle irreversible hydrocolloids essential to reduce the risks from the inhalation of toxic particles, such as using masks, keeping the work environment clean and with adequate ventilation.

The present study provides evidence for the need for quality control of Brazilian irreversible hydrocolloids, since contamination becomes a public health problem.

## Figures and Tables

**Table 1:** Analytical results of the amount of Li, Be, B, Na, Mg, Al, Si, P, K and Ti in irreversible hydrocolloid.

Analytical Results								
Contract Items	Irreversible Hydrocolloid 1	Irreversible Hydrocolloid 2	Irreversible Hydrocolloid 3	Irreversible Hydrocolloid 4	Irreversible Hydrocolloid 5	Irreversible Hydrocolloid 6	Irreversible Hydrocolloid 7	Irreversible Hydrocolloid 8
Li	<0.104 ppb	0.155 ppb	<0.104 ppb	<0.104 ppb	0.124 ppb	<0.104 ppb	<0.104 ppb	0.104 ppb
Be	<0.125 ppb	<0.125 ppb	<0.125 ppb	<0.125 ppb	<0.125 ppb	<0.125 ppb	<0.125 ppb	<0.125 ppb
B	180 ppb	173 ppb	142 ppb	125 ppb	98 ppb	108 ppb	97 ppb	72 ppb
Na	1307 ppb	222,398 ppb	238 ppb	236 ppb	79,393 ppb	231 ppb	231 ppb	13 ppb
Mg	21 ppb	408,852 ppb	20 ppb	21 ppb	28,468 ppb	21 ppb	21 ppb	3 ppb
Al	22 ppb	544 ppb	21 ppb	19 ppb	540 ppb	20 ppb	20 ppb	2 ppb
Si	957 ppb	1767 ppb	803 ppb	777 ppb	1807 ppb	731 ppb	707 ppb	117 ppb
P	295 ppb	19,439 ppb	223 ppb	23 ppb	2100 ppb	74 ppb	28 ppb	202 ppb
K	66 ppb	279,055 ppb	59 ppb	58 ppb	352,141 ppb	57 ppb	55 ppb	21.126 ppb
Ti	5 ppb	131,774 ppb	5 ppb	5 ppb	182,614 ppb	7 ppb	5 ppb	2 ppb
Analytical methods used in Determinations								
ICP-MS: ICP-MS => 028, 029, 030, 031, 033, 034, 035, 036, 037, 038								

**Table 2:** Analytical results of the amount of Mn, Co, Ni, V, Zn, Rb, As, Fe, Cu and Sr in irreversible hydrocolloid.

Analytical Results								
Contract Items	Irreversible Hydrocolloid 1	Irreversible Hydrocolloid 2	Irreversible Hydrocolloid 3	Irreversible Hydrocolloid 4	Irreversible Hydrocolloid 5	Irreversible Hydrocolloid 6	Irreversible Hydrocolloid 7	Irreversible Hydrocolloid 8
Mn	2 ppb	797 ppb	2 ppb	1 ppb	38 ppb	2 ppb	1 ppb	Undefined
Co	1 ppb	1 ppb	1 ppb	1 ppb	1 ppb	1 ppb	1 ppb	Undefined
Ni	6 ppb	12 ppb	5 ppb	5 ppb	9 ppb	5 ppb	5 ppb	Undefined
V	Undefined	875 ppb	Undefined	Undefined	1654 ppb	Undefined	Undefined	0.015 ppb
Zn	22 ppb	25 ppb	21 ppb	21 ppb	575,658 ppb	21 ppb	21 ppb	5 ppb
Rb	Undefined	6 ppb	Undefined	Undefined	13 ppb	Undefined	Undefined	Undefined
As	Undefined	3 ppb	Undefined	Undefined	3 ppb	Undefined	Undefined	Undefined
Fe	70 ppb	11,764 ppb	70 ppb	63 ppb	12295 ppb	907 ppb	65 ppb	3 ppb
Cu	4 ppb	9 ppb	4 ppb	4 ppb	14 ppb	4 ppb	4 ppb	1 ppb
Sr	4 ppb	6643 ppb	4 ppb	4 ppb	3199 ppb	4 ppb	4 ppb	Undefined
Analytical methods used in Determinations								
ICP-MS: ICP-MS => 039, 040, 041, 042, 043, 044, 045, 046, 047, 048								

**Table 3:** Analytical results of the amount of Y, Zr, Nb, Mo, Ru, Cd, Sn, Sb, Ba and La in irreversible hydrocolloid.

Analytical Results								
Contract Items	Irreversible Hydrocolloid 1	Irreversible Hydrocolloid 2	Irreversible Hydrocolloid 3	Irreversible Hydrocolloid 4	Irreversible Hydrocolloid 5	Irreversible Hydrocolloid 6	Irreversible Hydrocolloid 7	Irreversible Hydrocolloid 8
Y	Undefined	2 ppb	Undefined	Undefined	1 ppb	Undefined	Undefined	0.044 ppb
Zr	4 ppb	23 ppb	4 ppb	4 ppb	24 ppb	4 ppb	4 ppb	Undefined
Nb	8 ppb	30 ppb	8 ppb	8 ppb	420 ppb	7 ppb	7 ppb	Undefined
Mo	3 ppb	26 ppb	2 ppb	3 ppb	44 ppb	2 ppb	2 ppb	Undefined
Ru	<0.004 ppb	<0.004 ppb	<0.004 ppb	<0.004 ppb	<0.004 ppb	<0.004 ppb	<0.004 ppb	<0.004 ppb
Cd	<0.009 ppb	<0.009 ppb	<0.009 ppb	<0.009 ppb	1 ppb	<0.009 ppb	<0.009 ppb	<0.009 ppb
Sn	76 ppb	30 ppb	73 ppb	73 ppb	43 ppb	71 ppb	72 ppb	2 ppb
Sb	1 ppb	2 ppb	1 ppb	1 ppb	5 ppb	1 ppb	1 ppb	Undefined
Ba	4 ppb	162 ppb	4 ppb	4 ppb	51 ppb	4 ppb	4 ppb	Undefined
La	Undefined	3 ppb	Undefined	Undefined	3 ppb	Undefined	Undefined	Undefined
Analytical methods used in Determinations								
ICP-MS: ICP-MS => 049, 050, 051, 052, 053, 054, 055, 056, 057, 058								

**Table 4:** Analytical results of the amount of Ce, Hg, Pb, Th and U in irreversible hydrocolloid.

Analytical Results								
Contract Items	Irreversible Hydrocolloid 1	Irreversible Hydrocolloid 2	Irreversible Hydrocolloid 3	Irreversible Hydrocolloid 4	Irreversible Hydrocolloid 5	Irreversible Hydrocolloid 6	Irreversible Hydrocolloid 7	Irreversible Hydrocolloid 8
Ce	Undefined	6 ppb	Undefined	Undefined	5 ppb	Undefined	Undefined	Undefined
Hg	Undefined	1 ppb	Undefined	1 ppb	1 ppb	Undefined	1 ppb	Undefined
Pb	3 ppb	5 ppb	3 ppb	3 ppb	354 ppb	3 ppb	3 ppb	Undefined
Th	Undefined	1 ppb	Undefined	Undefined	1 ppb	Undefined	Undefined	Undefined
U	Undefined	1 ppb	Undefined	Undefined	1 ppb	Undefined	Undefined	Undefined
Analytical methods used in Determinations								
ICP-MS: ICP-MS => 059, 060, 061, 062, 063								
